# Association of Patient-Reported Outcomes with Hemophilia A Inhibitor Status and Treatment Product Type

**DOI:** 10.3390/jcm15041517

**Published:** 2026-02-14

**Authors:** Megan M. Ullman, Marilyn J. Manco-Johnson, Jonathan C. Roberts, Nicole Crook, Randall Curtis, Judith R. Baker, Joanne Wu, Michael B. Nichol

**Affiliations:** 1 Gulf States Hemophilia & Thrombophilia Center, University of Texas Health Science Center at Houston, Houston, TX 77030, USA; megan.ullman@sbcglobal.net; 2 Hemophilia & Thrombosis Center, University of Colorado Anschutz Medical Campus, Aurora, CO 80045, USA; marilyn.manco-johnson@cuanschutz.edu; 3 Bleeding & Clotting Disorders Institute, Dills Family Foundation Center for Research at BCDI, Peoria, IL 61614, USA; jroberts@ilbcdi.org; 4 Departments of Pediatrics and Medicine, University of Illinois College of Medicine at Peoria, Peoria, IL 61605, USA; 5 The Center for Comprehensive Care & Diagnosis of Inherited Blood Disorders, Orange, CA 92868, USA; ncrook@cibd-ca.org (N.C.); judithbaker321@gmail.com (J.R.B.); 6 Factor VIII Computing, Berkeley, CA 94720, USA; factor8@sbcglobal.net; 7 Leonard D. Schaeffer Center for Health Policy and Economics, University of Southern California, Los Angeles, CA 90089, USA; qfw@usc.edu

**Keywords:** hemophilia A, health-related quality of life, bleeding, clotting factor, emicizumab

## Abstract

**Objectives:** We compared patient-reported outcomes (PROs) in persons with hemophilia A (PwHA) by inhibitor status and prescribed treatment products. **Methods:** Hematology Utilization Group VIII study enrolled PwHA aged ≥ 2 years to collect PRO data via surveys. A clinical chart review documented the hemophilic severity, inhibitor level and treatment regimen. PROs were compared across inhibitor status and prescribed treatment products. **Results:** Among 85 enrolled PwHA, 9 (10.6%) had active inhibitors, 22 (25.9%) had tolerized inhibitors, and 54 (63.5%) had no inhibitors. The no-inhibitor group was significantly older (mean: 29.3 ± 13.5 years) than the tolerized (16.3 ± 9.5 years) and active inhibitor (21.9 ± 19.1 years; *p* = 0.001) groups. A larger proportion of participants with active inhibitors (66.7%) and no inhibitors (53.7%) reported having bleeds in the previous month compared to those with tolerized inhibitors (22.7%, *p* = 0.02). After covariate adjustment for age and hemophilia severity, the tolerized inhibitor group showed the lowest estimated number of joint bleeds compared to those of the no inhibitor and active inhibitor groups (*p* = 0.08), and the highest EQ-5D index score (*p* = 0.09). Emicizumab users reported significantly fewer bleeds in the previous months than those who were prescribed standard or extended half-life factor VIII (33.3% vs. 58.6%, 64.3%, *p* = 0.04). **Conclusions:** Participants with active inhibitors experienced joint bleeding rates similar to those of participants without inhibitors, likely attributable to emicizumab use. Tolerized participants reported the fewest joint bleeds and highest quality-of-life scores, potentially reflecting younger age and possible greater prophylaxis adherence. Emicizumab was associated with lower bleed rates compared to standard or extended half-life factor VIII products.

## 1. Introduction

Persons with hemophilia A (PwHA) can enjoy both a longer life and better quality of life than ever before due to expanding medical knowledge, improved therapeutic agents, and advancement of comprehensive care treatment [1,2]. Despite these innovations, patients can still suffer from serious complications, such as arthropathy with accompanying joint pain, and inhibitor development, which negatively impact their health-related quality of life (HRQoL) and further increase the burden of care.

Development of an inhibitor historically has been one of the most medically serious and costly complications in hemophilia treatment before emicizumab received approval from the U.S. Food and Drug Administration [3]. Persons with severe hemophilia A are the most likely to develop inhibitors and are estimated to have an overall 25–40% lifetime risk of inhibitor development, compared to a 5–15% lifetime risk in those with moderate/mild hemophilia A [4]. Individuals who develop inhibitors are twice as likely to be hospitalized for a bleeding complication and also face a greater risk of death [4,5,6,7]. Historically, therapeutic options for individuals with inhibitors have been limited and demanding, with often suboptimal results [8,9].

The increased bleeding and arthropathy associated with inhibitor development are a major cause of acute and chronic joint pain, morbidity and reduced everyday function [10]. Ineffective management of bleeding increases the risk of adverse outcomes from severe bleeding, including intracranial and gastrointestinal hemorrhage, which can be life-threatening. HRQoL assessments of PwHA demonstrate that when bleeding control is limited by inhibitor development, mental and social health, as well as physical functioning, are diminished [11].

Hemophilia treatment, regardless of inhibitor status, requires a lifetime commitment of considerable resources on the part of providers, payers, and those with hemophilia. Medical management of PwHA with inhibitors is particularly complex, as individual responses to bypassing agents and immune tolerance induction (ITI) regimens are unpredictable compared to replacement therapy in non-inhibitor individuals [12]. Previous research has demonstrated that hemophilia inhibitors are associated with a significant healthcare burden [12,13,14,15]. The last decade has brought significant advancements in hemophilia A coagulation therapies. New treatment products now available include extended half-life (EHL) coagulation factors and the first non-factor replacement antihemophilic medication, emicizumab. PwHA inhibitors using emicizumab from the HAVEN 1 study recorded meaningful improvements in their HRQoL and significantly reduced annualized bleeding rates [16,17,18].

Many burden-of-illness analyses excluded individuals with inhibitors due to this group’s unusually high healthcare utilization and costs. Since 1995, the Hematology Utilization Group Studies (HUGS) has collected data from hemophilia treatment centers (HTCs) to evaluate the costs of care and disease burden in PwH [19,20,21,22]. However, these studies collected data from the general hemophilia sample of individuals 2–64 years old, with small samples of participants with an inhibitor, which were usually excluded from the cost analyses. HUGS VIII focused specifically on the inhibitor status of PwHA to examine the costs and burden of hemophilia care, including the HRQoL, arthropathy, economic and psychosocial impacts on PwHA. This article reports data on patient-reported outcomes (PROs), including joint health and HRQoL. We also compared PROs by inhibitor status, and by the type of treatment product prescribed.

## 2. Material and Methods

### 2.1. Design

HUGS VIII is an observational cohort study that collected data from four large U.S. HTCs providing care to PwHA in Colorado, Texas, Illinois, and California. Inclusion criteria included the following: (1) age ≥ 2 years; (2) factor VIII activity level < 5%; (3) the participant received the majority of their hemophilia care at the HTC at least one year prior to enrollment to ensure adequate data collection throughout the study period; (4) for the inhibitor group, we used the inhibitor definition from Walsh [7] and included participants who had: (a) an inhibitor titer >1 Bethesda unit (BU) recorded at any HTC visit and/or (b) clinical records indicating that the participant received immune tolerance therapy (ITT) at any HTC visit; (5) the participant spoke either English or Spanish; and (6) the participant was willing to provide written informed consent. Individuals judged by the clinician to be cognitively impaired or those with any additional bleeding disorder were excluded. We enrolled participants with and without hemophilia A inhibitors at a 1:2 ratio.

### 2.2. Recruitment and Procedures

Eligible participants were identified by the site study coordinator during clinic visits or through clinical chart review. After obtaining informed consent, surveys were administered at baseline (enrollment) and 12 months later. Surveys were collected via RedCap, a secure web application. Participants received a $20 gift card for completing each survey. Recruitment began in April 2019, and data collection was completed in February 2022.

At each HTC, the study coordinator abstracted clinical information from participants’ medical charts for the period of six months prior to and 12 months after enrollment. The chart review collected information on the clinical characteristics of the participant, including the diagnostic level of factor VIII, inhibitor titer, treatment of inhibitor, treatment regimen, immune tolerance induction (ITI) status, history of joint procedures and pain medication prescription. Factor prescription records were reviewed for product class, including plasma-derived, standard and extended half-life recombinant factor VIII, emicizumab (Genentech Inc.—a member of the Roche Group, South San Francisco, CA, USA), and bypassing agents.

### 2.3. Measures

The baseline survey collected sociodemographic data, which included marital status, employment, education level, health insurance status and household income. We also collected data on personal characteristics, including health status, bleeding frequency, pain, joint involvement and stiffness impact, and hemophilia treatment. The post-12-month enrollment follow-up survey collected similar data, with the exception of sociodemographic information.

#### 2.3.1. Comparison Groups

Two types of comparison groups were defined based on the study objectives. First, based on hemophilia inhibitor status, participants were classified into three groups: (1) active inhibitors (those received a treatment appropriate for a positive factor VIII inhibitor with inhibitor titers > 1.0 BU six months prior to enrollment), (2) tolerized inhibitors (those with history of inhibitor titers > 1.0 BU plus past successful immune tolerance induction (ITI) and/or use of factor VIII for prophylaxis at enrollment), and (3) no inhibitors (those with history of inhibitor titers always ≤ 1.0 BU). Second, participants were classified based on prescription records from the chart review for the period of six months prior to and 12 months post-enrollment for plasma-derived, standard half-life (SHL) or EHL factor products, bypassing agents, or emicizumab. Participants were classified into three groups: (1) those prescribed plasma-derived products, SHL products or bypassing agents; (2) those prescribed only EHL products, or a combination of either plasma-derived products or SHL products with EHL products; or (3) those prescribed emicizumab at least once.

#### 2.3.2. Bleeding and Pain

Patient surveys documented self-reported bleeding events from the month prior to the baseline survey and during the three months prior to the follow-up survey. Participants reported whether they experienced joint or non-joint bleeds, and the number of each. They also reported whether they had a target joint, defined as experiencing four or more bleeds in a single joint in the six months prior to the baseline survey. Bleeding-related pain and chronic pain levels in general were measured by asking participants to specify the intensity of their pain using a visual analogue scale (VAS) ranging from 0 (no pain) to 10 (worst pain) [23].

#### 2.3.3. Joint Health

The self-reported joint pain and range-of-motion (ROM) limitation were obtained from two multiple-choice questions [20]. A five-item questionnaire on the stiffness impact was included and scored [24]. The score was transformed to a T-score, which is standardized to have a mean of 50 and a standard deviation (SD) of 10. Higher scores represent a more favorable status for the stiffness impact [24]. A medical chart review obtained information on participants who underwent any joint procedures for six months prior to enrollment.

#### 2.3.4. Health-Related Quality of Life

HRQoL was measured using the EuroQoL EQ-5D-3L [25]. The EQ-5D-3L consists of the EQ-5D descriptive system and the EQ-5D visual analogue scale (EQ VAS). The EQ-5D-3L descriptive system comprises five dimensions: mobility, self-care, usual activities, pain/discomfort and anxiety/depression. Each dimension has three levels: no problems, some problems, and extreme problems. The VAS records the respondent’s self-rated health on a vertical numeric scale ranging from 0 (the worst possible health status) to 100 (the best possible health status). The U.S.-based valuation algorithm was used to generate a time trade-off (TTO) index score, which is anchored at 0 for death and 1 for perfect health [26].

### 2.4. Statistical Analysis

Descriptive statistics, such as frequencies and proportions (for categorical variables) and means and standard deviations (for continuous variables), describe the sample in terms of the participant demographic and clinical characteristics, self-reported pain and HRQoL. Comparisons of individuals’ characteristics were made among three inhibitor groups or prescription groups using one-way ANOVA for continuous variables and chi-square tests for categorical variables. Given the modest sample size and the rarity of hemophilia A with inhibitors, this study was not formally powered to detect statistically significant differences between groups. Therefore, all between-group comparisons should be considered exploratory.

To compare the variables among the three inhibitor groups and three prescribed product groups, we defined clinically relevant differences among the groups as ≥5% absolute difference for categorical variables; 0.05-point difference for scores ranging on a 0–1 scale (e.g., EQ-5D index); 0.5-point difference for scores on a 0–10 scale (e.g., pain VAS); or 5-point difference for scores on a 0–100 scale (e.g., EQ VAS). These thresholds correspond to at least a small effect size (Cohen’s d ≥ 0.2) [27] and align with published minimally important differences (MIDs) where available (e.g., 0.040–0.074 for the EQ-5D index in hemophilia populations) [27,28]. These cut-points were used to complement the *p* values, given the limited statistical power of this study.

Missing data was excluded from the analyses on a complete-case basis. No imputation was performed due to the modest amount of missing data and the observational nature of this study. The proportion of missing values was low (<5% for most variables) and did not differ systematically by group.

Generalized linear regression models were used to assess the mean covariate-adjusted scores for pain and EQ-5D scores by inhibitor group and prescribed treatment group. These scores were obtained from general linear regression model least squares means, which were computed for each group, appropriately adjusted for the covariate effect in the general linear models. Covariates included age, hemophilic severity, and prophylactic treatment. The adjusted estimates represent the expected outcome for each comparison group if all participants had the same average age and severity. The negative adjusted means for bleed counts reflect values below the overall sample mean after adjustment and should be interpreted relative to the other groups rather than as literal negative counts.

## 3. Results

### 3.1. Participant Group Characteristics

Baseline data were collected from 85 consented PwHA. Sixty-seven participants (79%) completed the 12-month follow-up survey. Participant characteristics are summarized in Table 1 (demographics) and Table 2 (clinical characteristics).

Among the 85 participants, 9 (10.6%) had active inhibitors, 22 (25.9%) had tolerized inhibitors, and 54 (63.5%) had no inhibitors. The mean age was 24.8 ± 14.0 (standard deviation) years old, and the age ranged from 2.8 to 65.1 with a median of 23.2 years; 67.1% were adults 18 years or older. The mean age of the no-inhibitor group (28.7 ± 13.1) was older than those of the tolerized inhibitor group (16.3 ± 9.5 years) and active inhibitor group (21.9 ± 19.1) (*p* = 0.001). Children were more likely to be in the tolerized (59.1%) or active inhibitor (44.4%) groups than in the no inhibitor group (20.4%, *p* = 0.004). Adult participants or parents of pediatric participants with tolerized inhibitors (70.0%) were more likely to be married or with a partner than those with active inhibitors (44.4%) or no inhibitors (46.3%) (*p* = 0.17). Although there was no statistically significant difference in hemophilia healthcare treatment limitation related to inhibitor status, 19.5% and 25.0% higher proportions of participants with active inhibitors reported that their health insurance limited hemophilia treatment compared to those with no inhibitors or tolerized inhibitors (25.0% vs. 6.5%, 0%, *p* = 0.07) (Table 1).

When we classified participants based on their prescribed treatment products, 29 (34.1%) participants had plasma-derived or SHL prescriptions (two were prescribed bypassing agents), 14 (16.5%) had EHL prescriptions, and 42 (49.4%) were prescribed emicizumab. The mean age of participants treated with emicizumab was significantly younger than those of participants with SHL or EHL prescriptions (20.9±13.3 years vs. 29.5 ± 14 and 26.5 ± 13.4, respectively, *p* = 0.03, Table 1).

There were no significant differences in the hemophilia severity among the three inhibitor groups: active, tolerized and no inhibitor (100%, 100%, 83.3%, *p* = 0.17, respectively). There were also no significant differences in the hemophilia severity related to the three product class prescribed (*p* = 0.51).

Prophylaxis use was numerically higher in the active-inhibitor and tolerized inhibitor groups but was not statistically different across the inhibitor groups (*p* = 0.16).

Emicizumab was prescribed to a significantly higher proportion of participants with active inhibitors (88.9%) compared to the tolerized (59.1%) or no inhibitor (38.9%) (*p* = 0.02) groups.

### 3.2. Self-Reported Bleeding at Baseline

At baseline, participants with either active or no inhibitors were more likely to report having bleeds in the last month than those with tolerized inhibitors (66.7%, 53.7% and 22.7%, respectively, *p* = 0.02) (Table 3). Although the three inhibitor groups had no statistically significant differences in their covariate-adjusted mean numbers of bleeds in the last month, the active-inhibitor and no-inhibitor groups had numerically higher mean covariate-adjusted numbers of bleeds (0.66 ± 0.56, 0.88 ± 0.24 and 0.13 ± 0.40, respectively, *p* = 0.16). The proportion reporting any bleeds was significantly lower among emicizumab users (33.3%) compared to those prescribed SHL (58.6%) or EHL (64.3%; *p* = 0.04).

The proportions reporting joint bleeds in the previous month were significantly higher in the active inhibitor and no inhibitor groups than in the tolerized inhibitor group (44.4%, 35.2% vs. 4.5%, respectively, *p* = 0.01). The mean numbers of joint bleeds in the previous month were significantly higher in the active-inhibitor (0.56 ± 0.73) and no-inhibitor (0.57 ± 0.94) groups than that in the tolerized-inhibitor group (0.05 ± 0.21, *p* = 0.03). After covariate adjustment for age and hemophilia severity, the tolerized-inhibitor group had the lowest estimated mean number of joint bleeds compared to those of the other groups (*p* = 0.08; exploratory finding). Non-joint bleeds showed no significant differences across inhibitor or treatment groups.

Target joint bleeding (more than four bleeds in a single joint during the last six months) was observed more frequently among the active-inhibitor group (17.4%) and no-inhibitor group (8.4%) than among the tolerized-inhibitor group (*p* = 0.37) (Table 3).

The emicizumab group reported a numerically higher rate of target joint bleeding in the past 6 months than either the EHL or SHL groups (20.0% vs. 0% and 6.9%, respectively, *p* = 0.08, Table 3), although the differences were not statistically significant.

### 3.3. Self-Reported Joint Health and Pain at Baseline

Participants with tolerized inhibitors reported significantly lower mean joint stiffness T-scores (27.5 ± 5.1) compared to those with active (37.6 ± 12.9) or no inhibitors (33.3 ± 7.7, *p* = 0.002). Individuals with tolerized inhibitors reported 23.3% and 21.4% numerically higher rates for pain (54.5% vs. 77.8%, 75.9%, *p* = 0.16) and 12.2% and 14.0% higher rates for joint limitation of motion (54.5% vs. 66.7%, 68.5%, *p* = 0.51) compared to the active- or no-inhibitor groups.

The stiffness T-scores were not significantly different among the treatment product groups (*p* = 0.97). However, the SHL and emicizumab prescription groups had 20% and 16% numerically lower rates of self-reported joint pain (65.5%, 69.0% vs. 85.7%, *p* = 0.38), and 23.6% and 26.2% lower rates of joint range-of-motion limitation (62.1%, 59.5% vs. 85.7%, *p* = 0.19, exploratory finding), than those of the EHL group. The chronic pain scores were also significantly lower in the tolerized-inhibitor group (0.95 ± 1.53) compared to those in the active- (2.33 ± 2.83) or no-inhibitor (2.96 ± 2.62; *p* < 0.01) groups.

The self-reported joint pain and range-of-motion limitation showed no significant differences across inhibitors or treatment groups.

### 3.4. Health-Related Quality of Life at Baseline

The mean EQ-5D index score was significantly higher in the tolerized inhibitor group compared with those in the active or no inhibitor groups (0.92 ± 0.11 vs. 0.71 ± 0.24, 0.82 ± 0.22 *p* = 0.02). After covariate adjustment, the tolerized group had the highest estimated EQ-5D index score (*p* = 0.09; exploratory finding).

The active-inhibitor group reported a significantly worse mean EQ VAS than those of the tolerized and no inhibitor groups (68.33 ± 12.99, 85.64 ± 16.71, 82.34 ± 14.54, respectively, *p* = 0.02). After adjustment, the active-inhibitor group had the lowest EQ VAS score (*p* = 0.01). The HRQoL scores showed no significant differences among the treatment product groups.

### 3.5. Follow-Up Survey at 12 Months

At follow-up (n = 67), findings were largely consistent with those at baseline (Table 4). The active inhibitor and no inhibitor groups reported 31.2% or 14.5% of the numerically higher rates for self-reported bleeds in the previous three months than the tolerized group (*p* = 0.37). The active and no inhibitor groups also suggested greater mean covariate adjusted bleeds in the last three months than those in the tolerized group (0.52 ± 0.69, 0.88 ± 0.29, and 0.25 ± 0.47, respectively, *p* = 0.35). The active inhibitor group reported a numerically higher mean number of joint bleeds in the previous 3 months (1.17 ± 0.98) compared to the no inhibitor (0.50 ± 0.88) and tolerized (0.12 ± 0.33; *p* = 0.02) groups. After covariate adjustment, no significant differences were observed (*p* = 0.30; exploratory).

The tolerized inhibitor group continued to report numerically lower stiffness scores (26.3 ± 3.6 vs. 35.3 ± 10.5 active inhibitors and 33.1 ± 6.9 no inhibitors, *p* = 0.001), though this was not significant after adjustment (*p* = 0.41). The EQ-5D index scores remained the numerically highest in the tolerized group (0.94 ± 0.16) compared to those of the active inhibitor (0.76 ± 0.20) and no-inhibitor groups (0.84 ± 0.18; *p* = 0.06), with no significant differences after adjustment (*p* = 0.42).

The emicizumab group reported 8.1% and 18.1% of numerically lower bleeding rates than those of the SHL and EHL groups, respectively (Table 4), though this was not statistically significant (*p* = 0.58).

These exploratory findings suggest that the patterns observed at baseline persisted at 12 months, with tolerized inhibitor patients generally reporting the most favorable outcomes and active inhibitor patients on emicizumab showing bleeding and joint outcomes comparable to those in the no-inhibitor group. All between-group comparisons should be interpreted cautiously as exploratory, given the modest sample size and lack of formal powering for statistical significance.

## 4. Discussion

HUGS VIII was designed to evaluate the physical, social and economic burden of inhibitors in PwHA. However, at the time the study was initiated, a new non-factor therapy, emicizumab, became available on the market and was rapidly utilized by the inhibitor population. Using emicizumab, bleeding rates were significantly decreased in PwHA and active inhibitors, both in the pivotal studies [16,17,18] as well as in real-life practice. However, other real-world studies have suggested similar bleed rates in individuals without inhibitors on prophylaxis with factor VIII concentrates or emicizumab [29,30]. The current study showed positive outcomes for patients with hemophilia and inhibitors while using emicizumab (although persons using emicizumab were significantly less likely to report bleeding at baseline). Emicizumab was preferentially prescribed to patients with active inhibitors (88.9% of this group), likely to reflect indication bias toward those with a more severe historical bleeding phenotype. Despite this, emicizumab users reported significantly lower overall bleed rates at baseline, highlighting emicizumab’s effectiveness even in higher risk patients. In the post emicizumab era, long-term follow-up of both tolerized and active inhibitor patients will be critical to assess the joint health preservation, adherence to prophylaxis (in tolerized patients), and potential late complications. Tolerized patients may benefit from continued intensive prophylaxis to maintain early gains in joint protection, while active-inhibitor patients on emicizumab may require ongoing multidisciplinary support for musculoskeletal health, even with reduced bleeding.

Compared to people with tolerized inhibitors or no history of inhibitors, persons with active inhibitors showed no difference in the number of bleeding events, and no difference in joint pain or joint range-of-motion limitations. However, people with active inhibitors reported a higher rate of healthcare therapy limitations due to health insurance restrictions.

Immune tolerance was first widely used in the U.S. in the 1990s and 2000s. Immune tolerance regimens were successful in 70–80% of PwHA and inhibitors but the regimens were costly and very demanding of patients and families. In this study, people with tolerized inhibitors had better outcomes than those with no history of inhibitors, including (1) better joint health (less pain, joint stiffness or range-of-motion limitation), (2) less chronic pain, and (3) better HRQoL compared to the active- or no-inhibitor groups. The better outcomes observed in the tolerized inhibitor group compared to the no inhibitor group might be due to the younger age of the tolerized-inhibitor group compared to the inhibitor status cohort. Moreover, a higher proportion of the tolerized group used a prophylactic factor regimen than did the no inhibitor group. Inhibitor development among PwHA often occurs early in life when treatment is supervised by parents. The requirement of ITI for indefinite frequent factor VIII exposure and the motivation of parents and patients to avoid inhibitor recurrence may have encouraged better adherence to prophylaxis, yielding better joint outcomes than those among persons with hemophilia not complicated by inhibitor formation.

Since emicizumab received FDA approval and subsequently became widely available, bleeding rates, the primary determinate of outcomes in hemophilia, have been equalized between inhibitor and non-inhibitor patients. We are entering an era when the primary outcome of hemophilia is determined by the efficacy of novel therapeutics and is not anchored by disease severity or inhibitor status.

This study has several important limitations. First, the sample size was modest (n = 85 participants at baseline, with only 67 participants completing the one year follow-up survey), which substantially limited the statistical power to detect significant between-group differences for many study variables. As hemophilia A is a rare congenital condition, recruiting a large, representative sample remains inherently challenging. Second, the study sample was skewed toward a younger mean age in the tolerized inhibitor group (16.3 ± 9.5 years) compared to the no inhibitor group (29.3 ± 13.5 years) and active-inhibitor group (21.9 ± 19.1 years; *p* = 0.001). A higher proportion of children in the tolerized inhibitor group used prophylaxis, which made it important to adjust for age and treatment regimen in these analyses due to the age imbalance introducing a significant confounding variable for analysis. The better bleeding outcomes and higher EQ-5D scores observed in the tolerized inhibitor group may largely reflect the widespread adoption of modern intensive prophylaxis regimens (often initiated early in life) rather than the effect of successful immune tolerance induction (ITI) itself. Younger patients are also more likely to adhere to prophylaxis, have less cumulative joint damage, and engage in different activity levels, all of which could independently contribute to improved outcomes. Although age was included as a covariate in adjusted models, the modest sample size limited our ability to perform a detailed stratification of pediatric age groups (e.g., 2–5, 6–11, and 12–17 years), which would have been necessary to more fully evaluate age-related effects from inhibitor status or treatment effects. Additionally, the study sample included a higher proportion of children in the tolerized inhibitor group and a 12.2% higher proportion of participants using prophylaxis compared to the no-inhibitor group, further underscoring the need for cautious interpretation of between-group comparisons despite covariate adjustment. Although prophylaxis status was included as a covariate in adjusted models, residual confounding may persist due to the small sample size and potential collinearity with age and inhibitor status. Complete adjustment for prophylaxis intensity, adherence, and duration was not possible. Third, participants lost to follow-up (18 participants did not complete the one-year survey) further reduced the effective sample size and statistical power. However, this is an observational study without intervention, and we did not evaluate the PROs of changes between follow-up and baseline. We performed a separate sensitivity analysis restricted to participants who completed both the baseline and follow-up surveys, which yielded qualitatively similar results. Fourth, potential selection bias may exist. Participants were enrolled from a limited number of hemophilia treatment centers and may not fully represent the broader population of persons with hemophilia A, particularly those with more severe disease, less access to care, or who declined participation. The observational nature of this study also means that the treatment assignment (e.g., emicizumab vs. factor VIII products) was not randomized, and unmeasured confounders such as adherence, socioeconomic factors, or center specific practices could influence outcomes. Fifth, data on the time since successful immune tolerance induction and historical joint disease severity (e.g., cumulative joint bleeds prior to tolerization or baseline imaging scores) were not systematically collected. These factors could have influenced the current joint outcomes and represent an important unmeasured confounder. Finally, the COVID-19 pandemic significantly disrupted recruitment and data collection during the study period. Lockdowns, clinic closures, and reduced in person visits likely contributed to the lower-than-planned enrollment and higher rates of loss to follow-up, potentially biasing the sample toward participants with better access to care or more stable disease management. Given these limitations, we emphasize the importance of interpreting the findings with caution and focusing on clinically meaningful differences rather than relying solely on statistical significance.

## 5. Conclusions

These exploratory findings suggest that, in the era of emicizumab, bleeding rates and joint outcomes in patients with active inhibitors may approach those of non-inhibitor patients on prophylaxis. Tolerized patients demonstrated particularly favorable outcomes, potentially reflecting early intensive prophylaxis. Larger longitudinal studies are needed to confirm these emerging trends and fully characterize long term outcomes in the modern treatment landscape. Future studies should incorporate longitudinal analysis with repeated PRO assessments and a finer stratification of pediatric subgroups to better elucidate age specific effects and track within-subject trajectories.

## Figures and Tables

**Table 1:** Participants’ social demographic characteristics from initial survey.

Variable	Total N = 85	Inhibitor Status			*p* Value *	Prescription			*p* Value *
		Active Inhibitor N = 9, 10.6%	Tolerized Inhibitor N = 22, 25.9%	No Inhibitor N = 54, 63.5%		SHL † N = 29, 34.1%	EHL/EHL and SHL N = 14, 16.5%	EMI N = 42, 49.4%	
**Mean (SD) age**	24.8 (14.0)	21.9 (19.1)	16.3 (9.5)	28.7 (13.1)	0.001	29.5 (14.1)	26.5 (13.4)	20.9 (13.3)	0.03
**Age group**					0.004				0.08
Child	28 (32.9)	4 (44.4)	13 (59.1)	11 (20.4)		5 (17.2)	5 (35.7)	18 (42.9)	
Adult	57 (67.1)	5 (55.6)	9 (40.9)	43 (79.6)		24 (82.8)	9 (64.3)	24 (57.1)	
**Marital status**					0.17				0.75
Married/with a partner	43 (51.8)	4 (44.4)	14 (70.0)	25 (46.3)		16 (55.2)	8 (57.1)	19 (47.5)	
Single/not with a partner	40 (48.2)	5 (55.6)	6 (30.0)	29 (53.7)		13 (44.8)	6 (42.9)	21 (52.5)	
**Employment**					0.49				0.22
Employed	64 (76.2)	7 (87.5)	15 (68.2)	42 (77.8)		20 (69.0)	13 (92.9)	31 (75.6)	
Not employed	20 (23.8)	1 (12.5)	7 (31.8)	12 (22.2)		9 (31.0)	1 (7.1)	10 (24.4)	
**Income**					0.74				0.96
$75,000 or less	42 (57.5)	5 (71.4)	10 (55.6)	27 (56.3)		14 (58.3)	7 (53.8)	21 (58.3)	
More than $75,000	31 (42.5)	2 (28.6)	8 (44.4)	21 (43.8)		10 (41.7)	6 (46.2)	15 (41.7)	
**Health insurance limits hemophiliac treatment**	4 (7.0)	2 (25.0)	0 (0.0)	2 (6.5)	0.07	0 (0.0)	1 (16.7)	3 (9.4)	0.28

Notes: Data were presented as numbers (column percentages) for categorical variables or means (standard deviations) for continuous variables. * *p* values were calculated from chi-square tests for categorical variables and from one-way ANOVA for continuous variables. † Two participants were prescribed only bypassing agents. Abbreviations: N, number; SHL, standard half-life coagulation factors; EHL, extended half-life coagulation factors; EMI, emicizumab; SD, standard deviation; $, US dollars

**Table 2:** Clinical characteristics from clinical chart review.

Variable	Total N = 85	Inhibitor Status			*p* Value *	Prescription			*p* Value *
		Active Inhibitor N = 9, 10.6%	Tolerized Inhibitor N = 22, 25.9%	No Inhibitor N = 54, 63.5%		SHL † N = 29, 34.1	EHL/EHL and SHL N = 14, 16.5%	EMI N = 42, 49.4%	
**Hemophilia severity**					0.17				0.51
Moderate	10 (11.8)	0 (0.0)	1 (4.5)	9 (16.7)		5 (17.2)	1 (7.1)	4 (9.5)	
Severe	75 (88.2)	9 (100.0)	21 (95.5)	45 (83.3)		24 (82.8)	13 (92.9)	38 (90.5)	
**Mean age at which factor use was started (SD)**	4.9 (9.3)	6.9 (10.6)	6.7 (14.4)	3.8 (5.7)	0.37	4.9 (8.4)	7.1 (17.7)	4.1 (5.1)	0.59
**Prophylactic treatment**	74 (88.1)	9 (100.0)	21 (95.5)	44 (83.0)	0.16	21 (72.4)	14 (100.0)	39 (95.1)	<0.01
**Prophylactic treatment in severe patients (N = 75)**	66 (88.0)	9 (100.0)	18 (85.7)	39 (86.7)	0.49	18 (75.0)	13 (100.0)	35 (92.1)	0.04
**Used bypassing agents**	7 (8.2)	6 (66.7)	1 (4.5)	0 (0.0)	<0.0001	1 (3.4)	0 (0.0)	6 (14.3)	0.12
**Received ITI treatment**	26 (30.6)	6 (66.7)	20 (90.9)	0 (0.0)	<0.0001	6 (20.7)	1 (7.1)	19 (45.2)	0.01
**History of joint procedure**	1 (1.2)	1 (11.1)	0 (0.0)	0 (0.0)	0.01	0 (0.0)	0 (0.0)	1 (2.4)	0.6
**Used opioid pain medication**	8 (9.4)	1 (11.1)	1 (4.5)	6 (11.1)	0.66	4 (13.8)	2 (14.3)	2 (4.8)	0.35
**Used non-opioid pain medication**	19 (22.4)	3 (33.3)	4 (18.2)	12 (22.2)	0.65	7 (24.1)	1 (7.1)	11 (26.2)	0.32

Notes: Data were presented as numbers (column percentages) for categorical variables or means (standard deviations) for continuous variables. * *p* values were calculated from chi-square tests. † Two participants were prescribed only bypassing agents. Abbreviations: N, number; SHL, standard half-life coagulation factors; EHL, extended half-life coagulation factors; EMI, emicizumab; SD, standard deviation. ITI, immune tolerance induction

**Table 3:** Self-reported bleeding, joint problems, and health-related quality of life from initial survey.

Variable	Total N = 85	Inhibitor Status			*p* Value *	Prescription			*p* Value *
		Active Inhibitor N = 9, 10.6%	Tolerized Inhibitor N = 22, 25.9%	No Inhibitor N = 54, 63.5%		SHL† N = 29, 34.1	EHL/EHL and SHL N = 14, 16.5%	EMI N = 42, 49.4%	
Self-reported bleeding									
Had bleeds in last month	40 (47.1)	6 (66.7)	5 (22.7)	29 (53.7)	0.02	17 (58.6)	9 (64.3)	14 (33.3)	0.04
Number of bleeds in last month (SD)	0.87 (1.36)	0.88 (0.83)	0.27 (0.55)	1.11 (1.57)	0.049	1.03 (1.32)	0.93 (0.83)	0.73 (1.53)	0.65
Covariate adjusted number of bleeds in last month (SE) †	NA	0.66 (0.56) a	0.13 (0.40) a	0.88 (0.24) a	0.15	0.70 (0.35) a	0.43 (0.49) a	0.54 (0.31) a	0.81
Had joint bleeds in last month	24 (28.2)	4 (44.4)	1 (4.5)	19 (35.2)	0.01	9 (31.0)	6 (42.9)	9 (21.4)	0.28
Numbers of joint bleeds in last month (SD)	0.44 (0.82)	0.56 (0.73)	0.05 (0.21)	0.57 (0.94)	0.03	0.52 (0.99)	0.43 (0.51)	0.38 (0.79)	0.79
Covariate adjusted numbers of joint bleeds in last month (SE) ‡	NA	0.37 (0.32) a	−0.09 (0.24) b	0.40 (0.15) a	0.08	0.31 (0.21)	0.12 (0.29)	0.25 (0.18)	0.76
Had non-joint bleeds in last month	26 (30.6)	2 (22.2)	4 (18.2)	20 (37.0)	0.23	11 (37.9)	6 (42.9)	9 (21.4)	0.18
Numbers of non-joint bleeds in last month (SD)	0.43 (0.81)	0.25 (0.71)	0.23 (0.53)	0.54 (0.91)	0.26	0.52 (0.74)	0.50 (0.65)	0.34 (0.91)	0.63
Covariate adjusted number of non-joint bleeds in last month (SE) ‡	NA	0.23 (0.35) a	0.22 (0.25) a	0.48 (0.15) a	0.49	0.37 (0.21) a	0.29 (0.30) a	0.27 (0.19) a	0.89
Had ≥4 bleeds in single joint in past 6 months	10 (12.0)	2 (22.2)	1 (4.8)	7 (13.2)	0.37	2 (6.9)	0 (0.0)	8 (20.0)	0.08
Joint problems									
Chronic pain level (SD)	2.39 (2.54)	2.33 (2.83)	0.95 (1.53)	2.96 (2.62)	<0.01	2.66 (2.48)	2.00 (1.62)	2.33 (2.85)	0.72
Covariate adjusted chronic pain level (SE) ‡	NA	1.62 (0.88) a,b	0.85 (0.66) b	2.18 (0.40) a	0.12	1.72 (0.56) a	0.91 (0.79) a	2.03 (0.50) a	0.29
Self-reported joint pain	60 (70.6)	7 (77.8)	12 (54.5)	41 (75.9)	0.16	19 (65.5)	12 (85.7)	29 (69.0)	0.38
Joint range-of-motion limitation	55 (64.7)	6 (66.7)	12 (54.5)	37 (68.5)	0.51	18 (62.1)	12 (85.7)	25 (59.5)	0.19
Stiffness score (SD)	32.2 (8.4)	37.6 (12.9)	27.5 (5.1)	33.3 (7.7)	0.002	32.5 (8.1)	31.8 (5.2)	32.2 (9.5)	0.97
Covariate adjusted stiffness score (SE) ‡	NA	35.69 (0.73) a,b	27.98 (2.03) a	30.67 (1.24) a	0.02	32.56 (1.56) a	30.68 (2.45) a	31.12 (1.74) a	0.60
Health-related quality of life									
EQ VAS (SD)	81.70 (15.59)	68.33 (12.99)	85.64 (16.71)	82.34 (14.54)	0.02	79.61 (18.21)	84.36 (10.06)	82.21 (15.37)	0.63
Covariate adjusted EQ VAS (SE) ‡	NA	70.21 (5.58) a	85.08 (4.14) b	86.23 (2.55) b	0.01	78.55 (3.57) a	82.29 (5.00) a	80.70 (3.18) a	0.70
EQ index score (SD)	0.83 (0.21)	0.71 (0.24)	0.92 (0.11)	0.82 (0.22)	0.02	0.87 (0.19)	0.81 (0.18)	0.82 (0.23)	0.58
Covariate adjusted EQ index score (SE) ‡	NA	0.77 (0.08) a,b	0.93 (0.06) a	0.88 (0.03) a	0.09	0.90 (0.05) a	0.84 (0.07) a	0.83 (0.04) a	0.36

Notes: Data were presented as numbers (column percentages) for categorical variables or means (standard deviations) for continuous variables. * *p* values were calculated from chi-square tests. † Two participants were prescribed only bypassing agents. ‡ Covariate adjusted means and standard errors were obtained from general linear regression model least squares means, which were computed for each inhibitor group or prescription product, appropriately adjusted for the covariate effect in the general linear models. For each row, least squares means with different letters (a, b) across groups (inhibitor status or prescribed products) were significantly different according to the Tukey multiple comparison procedure (*p* < 0.05). Covariates included age and hemophilia severity. Abbreviations: N, number; EQ VAS, EuroQol visual analogue scale; NA, not applicable; SHL, standard half-life coagulation factors; EHL, extended half-life coagulation factors; EMI, emicizumab, SD, standard deviation; SE, standard error.

**Table 4:** Self-reported bleeding, joint problems, and health-related quality of life from follow-up survey.

		Inhibitor Status				Prescription			
Variable	Total N = 67	Active Inhibitor N = 6, 9%	Tolerized Inhibitor N = 17, 25.4%	No Inhibitor N = 44, 65.7%	*p* Value *	SHL † N = 26, 38.8%	EHL/EHL and SHL N = 10, 14.9%	EMI N = 31, 46.3%	*p* Value *
Self-reported bleeding									
Had bleeds in last three months	32 (47.8)	4 (66.7)	6 (35.3)	22 (50.0)	0.37	13 (50.0)	6 (60.0)	13 (41.9)	0.58
Number of bleeds in last three months (SD)	1.15 (2.31)	1.17 (0.98)	1.24 (3.83)	1.11 (1.62)	0.98	1.46 (3.24)	1.70 (2.16)	0.71 (1.10)	0.34
Covariate adjusted number of bleeds in last three months (SE) ‡	NA	0.52 (0.69)	0.25 (0.47)	0.88 (0.29)	0.35	0.52 (0.38)	0.46 (0.59)	0.67 (0.38)	0.88
Number of joint bleeds in last three months (SD)	0.46 (0.82)	1.17 (0.98)	0.12 (0.33)	0.50 (0.88)	0.02	0.35 (0.80)	0.80 (1.03)	0.45 (0.77)	0.34
Covariate adjusted number of joint bleeds in last three months (SE) ‡	NA	0.09 (0.32)	0.01 (0.24)	0.34 (0.14)	0.30	0.07 (0.19)	0.08 (0.29)	0.27 (0.18)	0.54
Number of non-joint bleeds in last three months (SD)	0.69 (1.99)	0.00 (0.00)	1.12 (3.60)	0.61 (1.04)	0.46	1.12 (2.94)	0.90 (1.52)	0.26 (0.63)	0.25
Covariate adjusted number of non-joint bleeds in last three months (SE) ‡	NA	0.37 (0.49)	0.24 (0.31)	0.54 (0.19)	0.58	0.42 (0.25)	0.36 (0.39)	0.38 (0.25)	0.97
Had joint bleeds in last three months	20 (29.9)	4 (66.7)	2 (11.8)	14 (31.8)	0.04	5 (19.2)	5 (50.0)	10 (32.3)	0.18
Had non-joint bleeds in three last months	20 (29.9)	0 (0.0)	5 (29.4)	15 (34.1)	0.23	11 (42.3)	3 (30.0)	6 (19.4)	0.17
Joint problems									
Self-reported joint pain	44 (65.7)	4 (66.7)	8 (47.1)	32 (72.7)	0.17	18 (69.2)	8 (80.0)	18 (58.1)	0.40
Joint range-of-motion limitation	37 (55.2)	4 (66.7)	7 (41.2)	26 (59.1)	0.38	15 (57.7)	5 (50.0)	17 (54.8)	0.92
Stiffness score (SD)	31.6 (7.3)	35.3 (10.5)	26.3 (3.6)	33.1 (6.9)	0.001	31.4 (7.6)	31.0 (5.9)	31.9 (7.6)	0.94
Covariate adjusted stiffness score (SE) ‡	NA	31.0 (2.9)	28.3 (2.1)	30.8 (1.3)	0.41	29.7 (1.6)	28.7 (2.6)	31.7 (1.6)	0.30
Health-related quality of life									
EQ VAS (SD)	82.09 (15.87)	69.33 (16.51)	89.71 (10.58)	80.89 (16.32)	0.02	82.77 (13.58)	86.20 (13.10)	80.19 (18.42)	0.57
Covariate adjusted EQ VAS (SE) ‡	NA	76.3 (7.0)	85.7 (5.0)	85.7 (3.1)	0.35	80.8 (4.0)	85.0 (6.2)	82.0 (3.9)	0.76
EQ-5D index score (SD)	0.86 (0.18)	0.76 (0.20)	0.94 (0.16)	0.84 (0.18)	0.06	0.87 (0.19)	0.88 (0.11)	0.84 (0.20)	0.77
Covariate adjusted EQ index score (SE) ‡	NA	0.87 (0.09)	0.97 (0.07)	0.89 (0.04)	0.42	0.94 (0.05)	0.93 (0.08)	0.86 (0.05)	0.30

Notes: Data are presented as numbers (column percentages) for categorical variables or means (standard deviations) for continuous variables. * *p* values were calculated from chi-square tests for categorical variables and from one-way ANOVA for continuous variables. † Two participants were prescribed only bypassing agents. ‡ Covariate adjusted means and standard errors were obtained from general linear regression model least squares means, which were computed for each inhibitor group or prescription product, appropriately adjusted for the covariate effect in the general linear models. Covariates included age and hemophilia severity. Abbreviations: N, number; EQ VAS, EuroQol visual analogue scale; NA, not applicable; SHL, standard half-life coagulation factors; EHL, extended half-life coagulation factors; EMI, emicizumab; SD, standard deviation; SE, standard error.

## Data Availability

The data presented in this study are available upon request from the corresponding author, Michael B. Nichol (mnichol@usc.edu), due to ethical restrictions.
